# Experimental Method for Tensile Testing of Unidirectional Carbon Fibre Composites Using Improved Specimen Type and Data Analysis

**DOI:** 10.3390/ma14143939

**Published:** 2021-07-14

**Authors:** Rajnish Kumar, Lars P. Mikkelsen, Hans Lilholt, Bo Madsen

**Affiliations:** Division of Wind Energy Materials and Components, Department of Wind Energy, Technical University of Denmark, 4000 Roskilde, Denmark; lapm@dtu.dk (L.P.M.); hali@dtu.dk (H.L.); boma@dtu.dk (B.M.)

**Keywords:** carbon fibre composite, test specimen, static tensile testing, stress–strain curve, strain to failure

## Abstract

This paper presents an experimental method for tensile testing of unidirectional carbon fibre composites. It uses a novel combination of a new specimen geometry, protective layer, and a robust data analysis method. The experiments were designed to test and analyze unprotected (with conventional end-tabs) and protected (with continuous end-tabs) carbon fibre composite specimens with three different specimen geometries (straight-sided, butterfly, and X-butterfly). Initial stiffness and strain to failure were determined from second-order polynomial fitted stress–strain curves. A good agreement between back-calculated and measured stress–strain curves is found, on both composite and fibre level. For unprotected carbon composites, the effect of changing specimen geometry from straight-sided to X-butterfly was an increase in strain to failure from 1.31 to 1.44%. The effect of protection on X-butterfly specimens was an increase in strain to failure from 1.44 to 1.53%. For protected X-butterfly specimens, the combined effect of geometry and protection led to a significant improvement in strain to failure of 17% compared to unprotected straight-sided specimens. The observed increasing trend in the measured strain to failure, by changing specimen geometry and protection, suggests that the actual strain to failure of unidirectional carbon composites is getting closer to be realized.

## 1. Introduction

Carbon fibre composites are a unique material with exceptional mechanical properties and therefore widely utilized for structural applications in the aerospace, automotive, and wind energy sectors. The most basic mechanical properties important for the design and application of such materials are stiffness and strength. These mechanical properties are obtained from static tensile test of unidirectional (UD) fibre composites. Accurate determination of stiffness is challenging since the stress–strain curve is typically not well defined at the start of test, and it might also not be linear; especially for carbon fibre composites, the stress–strain curve is clearly non-linear [1,2]. It is also challenging to determine the actual strength since it requires that test specimens fail by tensile failure in the gauge section, away from the grips [3,4,5]. This is a challenge for all UD fibre composites, and it is even more difficult for high strength composites, such as carbon fibre composites. During tensile testing of a test specimen, tensile load in the specimen is introduced through shear load by the action of lateral gripping at the end regions of the specimen. For UD fibre composites, due to the alignment of fibres, shear strength is considerably lower than tensile strength. Thus, the test specimen is likely to fail in shear at the grip region due the introduced high shear stresses. Use of end-tabs increases thickness at the ends of the specimen, and this leads to reduction of shear stress in the composite. End-tabs also protect the specimen from being damaged by the serrated grip surfaces. However, stress concentration regions develop near the start of end-tabs due to the sudden change in thickness [6,7,8]. This is well known to act as failure initiation points, causing failure of the specimen at a stress level significantly lower than the actual strength of the composite material [9]. In the simple case of using end-tabs on straight-sided test specimens, it is well accepted that this leads to underestimation of the actual strength of UD composites [10].

One strategy to obtain failure of a test specimen away from the grips is to optimize the specimen geometry towards having specimens with non-straight sides [9,11,12]. Such specimens are denoted by different names, e.g., dogbone, dumbbell, and butterfly. The specimens are designed with curved sections of constant radius to smoothly reduce the width of the specimen towards a minimum at the middle where the specimen will experience the largest tensile stress. The specimens are designed to fail in the middle gauge section. Butterfly shaped specimens with a narrow-gauge section of constant rectangular shape have previously been used for testing of UD composites [12,13]. Korkiakoski et al. [13] carried out finite element analyses of such butterfly specimens with different curvatures and lengths to optimize the specimen geometry to have failure in the gauge section. Another strategy to obtain failure of a test specimen away from the grips is to use specimen protection (or continuous end-tabs). Czél et al. [14,15] were first to propose a specimen type with protective layers to suppress end-tab stress concentrations during tensile test. They performed experiments on thin UD carbon composites with thickness up to 0.116 mm, and reported a strain to failure of 1.88%, which was significantly higher than 1.50% obtained with conventional unprotected specimens with end-tabs. Tahir et al. [16] performed tensile testing of UD carbon composites with a thickness of 1.4 mm using aluminum protective layers. They reported an improvement of approximately 10% in failure stress due to the protective layer.

This paper presents advances in tensile testing of UD carbon fibre composites by using a novel specimen type and a robust data analysis method. The proposed specimen type is developed by combining the two strategies of specimen geometry and specimen protection. Results of tensile testing of unprotected and protected carbon fibre composite specimens with three different specimen geometries (straight-sided, butterfly, and X-butterfly) are presented and analyzed. The method of using protective layers requires an operational data analysis method to back-calculate the stress–strain curve of the central composite layer. A second-order polynomial fitting method is used to obtain practical operational stress–strain curves from which stiffness and strain to failure are determined. The efficacy of the data analysis method is demonstrated by comparing back-calculated stress–strain curves of protected carbon composites with measured stress–strain curves of unprotected carbon composites and single carbon fibres.

## 2. Materials and Methods

### 2.1. Protective Layer

The strain to failure of the protective layer (continuous end-tabs) should be higher than the composite being tested. This is to ensure the survival of protective layers until the failure of the composite, as the strain is measured on the surface of the protective layers. Therefore, in the present study, glass composite was selected as protective layer, having significantly higher strain to failure compared to carbon composite. Moreover, the protective layer should be thick enough to accommodate the high stresses around the edges of grip surfaces [14]. The thickness of the protective glass composite layer used in this study was selected the same as the end-tabs thickness of 2 mm, based on the ISO 527-5 test method [17].

UD glass/epoxy composite laminates (called G) for protective layers were manufactured with vacuum infusion using a quasi-unidirectional glass fibre non-crimp fabric (Uniaxial 1322, 1397 g/m^2^) supplied by Owens Corning, Belgium. The rovings of UD fibres were held in place by backing fibre rovings at ± 80°. The areal weights for UD and backing fibre rovings (called b) were 1322 and 60 g/m^2^, respectively. The used resin system was Araldite^®^ LY 1568 CH epoxy resin and Aradur^®^ 3489 CH hardener, supplied by Huntsman GmbH, Switzerland. The fabric stacking sequence for the composites was [b/0]_S_. The resin/hardener mix of 100:28 (parts by weight) was degassed before infusion. The infusion was performed perpendicular to the fibre direction under a vacuum pressure of 10 mbar. The infused fibre/matrix assembly was cured at 75 °C for 5 h, and then at 40 °C for 19 h. The area of the manufactured composite laminates was 1200 × 600 mm^2^, and with thickness in the range 2.1–2.2 mm.

### 2.2. Protected Carbon Composites

UD carbon/vinyl ester composite laminates (called C) were supplied by Fiberline Composites A/S, Denmark, in the form of pultruded profiles with a cross-sectional area of 105 mm × 1.9 mm. The carbon fibre type was 50 k PAN-based fibre (Mitsubishi Chemical Corporation, Tokyo, Japan).

Protected carbon composite laminates (G/C/G) were manufactured by sandwiching a layer of UD carbon composite between two layers of UD glass composites, with total thickness in the range 6.1–6.5 mm. First, the glass composite layers were mounted temporarily with clamps in the wanted position. Holes for guide pins were drilled at the diagonal position. Fixation with pins was found important to keep the layer in position during the bonding operations. The composite surfaces to be bonded were then slightly ground and cleaned with ethanol. A structural epoxy adhesive (3M, DP460) was applied on one surface of each of the two glass composite layers, and with some extra thickness in the middle to obtain a flow and thereby to avoid trapping of air bubbles. Similarly, the adhesive was applied on both sides of the carbon composite layer in a uniform layer. The composite layers were placed carefully in position using the guide pins and were pressed between two steel press plates using clamps. The adhesive was cured at room temperature for 24 h, followed by post-curing at 40 °C for 16 h.

### 2.3. Test Specimens

#### 2.3.1. Geometry and Tabs

**Figure 1:**
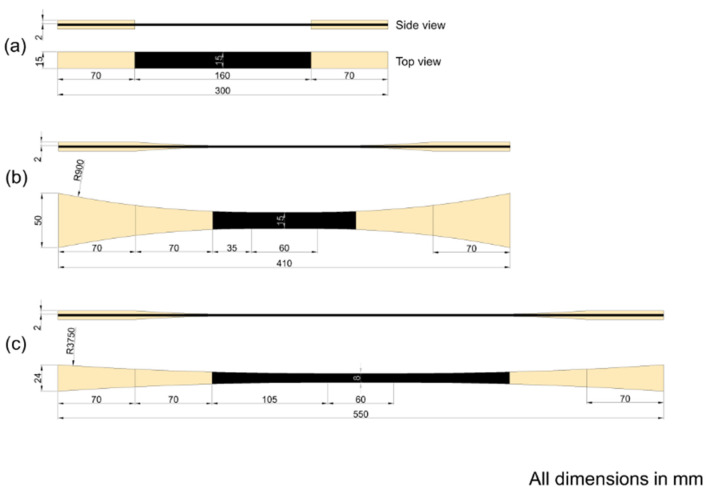
Unprotected test specimens; geometry and dimensions: (**a**) straight-sided, (**b**) butterfly, (**c**) X-butterfly.

Three different test specimen geometries were used in the study: (1) Rectangular shaped specimens, denoted *straight-sided*, see Figure 1a, based on well-known and widely used standards like ISO 527-5:2009 [17] and ASTM D3039 [18]. (2) Standard non-straight sided specimens, denoted *butterfly*, see Figure 1b, specially designed based on a previous study at DTU Wind Energy [13]. (3) Elongated non-straight sided specimens, denoted *X-butterfly* see Figure 1c, having a longer length and reduced curvature has been developed at DTU Wind Energy, and is presented here for the first time. All test specimen geometries have 70 mm long gripping regions from the ends of the specimen.

**Figure 2:**
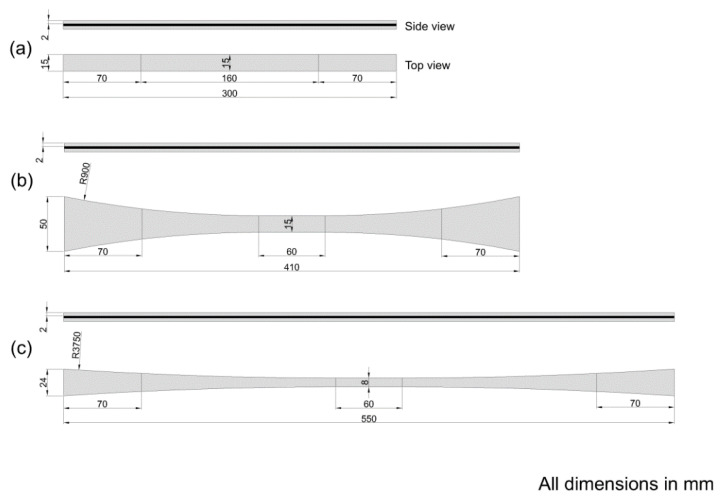
Protected test specimens; geometry and dimensions: (**a**) straight-sided, (**b**) butterfly, (**c**) X-butterfly.

For *unprotected* test specimens (C and G), biaxial glass composite was used as end-tabs (see Figure 1). Straight-edged end-tabs were used for the straight-sided specimens, and tapered end-tabs (tapered over 70 mm length) were used for the butterfly and X-butterfly specimens. For unprotected glass composites (G), only the straight-sided test specimen geometry was used. For *protected* test specimens (G/C/G), UD glass composite was used as protective layer (continuous end-tabs) (see Figure 2).

#### 2.3.2. Preparation

The straight-sided test specimens were obtained by using a circular saw with a carbide steel-tipped blade. The butterfly and X-butterfly test specimens were obtained by using a CNC milling machine. For the test specimens of unprotected composites, biaxial glass composite laminate (Electro-Isola A/S, Vejle, Denmark) with a thickness of 2 mm was used as end-tab material, with the fibres positioned at ±45° to the specimen long direction. The surfaces of the specimens at the end-tab areas were slightly ground using a disc sander and were cleaned with ethanol. The end-tabs were bonded to the specimens using a structural epoxy adhesive (3M, DP460) with a cure cycle of room temperature for 24 h and 40 °C for 16 h.

#### 2.3.3. Experimental Design

**Figure 3:**
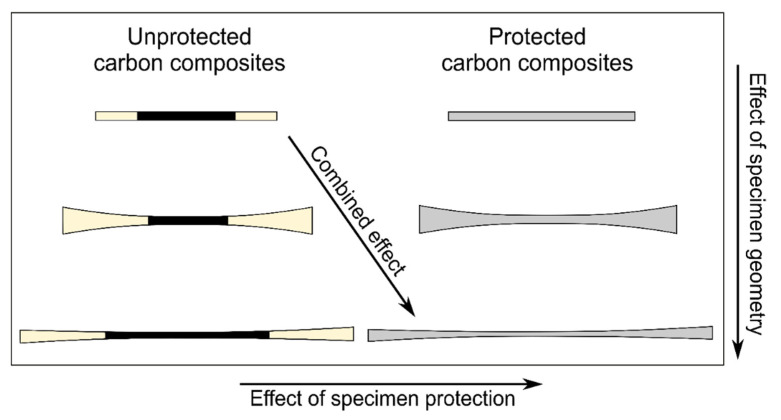
Schematic diagram representing the design of the experiments.

A schematic diagram representing the design of the experiments is shown in Figure 3. The unprotected and protected carbon composite specimens with the three different specimen geometries (straight-sided, butterfly, and X-butterfly) were analyzed. As shown in the diagram, the *effect of specimen geometry* was analyzed by comparing the different specimen geometries for composites with the same specimen protection. The *effect of specimen protection* was analyzed by comparing unprotected and protected carbon composite specimens with the same specimen geometry. Finally, the *combined effect of specimen geometry and protection* was analyzed by comparing the unprotected straight-sided carbon composite specimens with protected X-butterfly carbon composite specimens.

### 2.4. Composite Characterization

#### 2.4.1. Fibre Volume Fraction

The fibre volume fraction of the carbon composite was determined using the gravimetric method (ASTM D3171-15, ASTM D2584). Five samples with a rectangular area of 15 × 15 mm^2^ were cut from the composite laminates. The density of the samples was measured using the buoyancy method based on Archimedes’ principle. The fibre weight fraction of the samples was determined by the resin burn-off technique. The fibre volume fraction of the composite was calculated by standard Equations [19] using densities of carbon fibre, and matrix as 1.77, and 1.20 g/cm^3^, respectively. The density of carbon fibre was measured based on gas pycnometry method. The measured fibre volume fraction and porosity content were found to be 67 ± 1% and 0.7 ± 0.3%, respectively.

#### 2.4.2. Composite Volume Fractions

The volume fraction of carbon composite in the protected carbon composite specimens (G/C/G) was determined as follows:

(1) $$V_{c}=\frac{t_{C}}{t_{G/C/G}}$$

where V_C_, t_C_, and t_G/C/G_ are volume fraction of carbon composite, thickness of unprotected carbon composite layer, and thickness of protected carbon composite.

**Figure 4:**
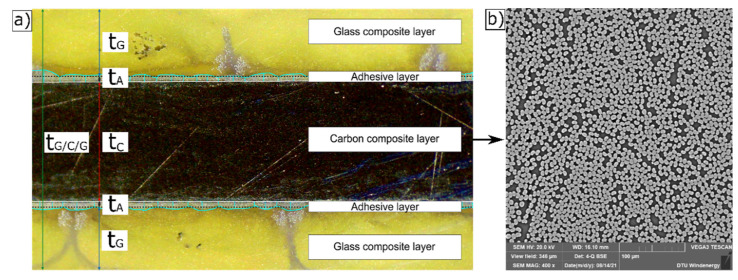
(**a**) Optical microscopy image of protected carbon composite specimen showing the three composite layers and the two adhesive layers between them, (**b**) representative SEM image showing microstructure of carbon composite layer.

To determine the thickness of the adhesive layer, samples of the protected carbon composites with a rectangular area of 25 × 25 mm^2^ were cut from the tab area of the tested specimens. The samples were cast into cylindrical blocks of epoxy resin. Edges of the blocks were ground and polished. By using an optical microscope (Leica S9i, Wetzlar, Germany), the thickness of the adhesive layer (t_A_) between the carbon and glass composite layers was measured using ImageJ software (see example in Figure 4a). The measured average thickness of glue layer based on two different samples of each protected specimen type was 0.14 ± 0.06 mm. Based on 10 sets of measurements on a same sample, the uncertainty of measurement was found to be 0.002 mm. The microstructure of carbon composite was examined using scanning electron microscope (Vega3 Tescan, Brno, Czech Republic) at 400× magnification. A uniform distribution of fibres was observed, with no sign of porosities (see Figure 4b), and the latter observation is supported by the measured low porosity fraction.

The volume fraction of the adhesive layer (V_A_) in the protected carbon composite was determined as follows:

(2) $$V_{A}=\frac{2t_{A}}{t_{G/C/G}}$$

Finally, the volume fraction of glass composite (V_G_) in the protected carbon composite was determined as follows:

(3) $$V_{G}=1-\left(V_{C}+V_{A}\right)=\frac{t_{G/C/G}-(t_{C}+2t_{A})}{t_{G/C/G}}$$

#### 2.4.3. Tensile Properties

Tensile testing of the composite specimens was performed on a universal testing machine (Instron 88R1331, Norwood, MA, USA) with 250 kN load cell for the unprotected and protected carbon composites, and 50 kN load cell for the unprotected glass composites. The crosshead speed was 2 mm/min for all specimens. Strain was measured with two 6 mm long single strain gauges (LY11-6/350, Darmstadt, Germany) placed on the center on each side of the test specimens. Six specimens each of all specimen types (unprotected and protected) were tested.

### 2.5. Stress–Strain Curve Analysis

#### 2.5.1. Second-Order Polynomial Fitting

A general observation is that the stress–strain curves of carbon composites curve upwards [1,2], and this indicates an increasing tangent stiffness with increasing strain. Markussen et al. [2] reported an almost linear relation between tangent stiffness and strain for unidirectional carbon composites, implying a second-order polynomial relation between stress (σ) and strain (ε).

A linear relation between tangent stiffness and strain can be expressed as:

(4) $$E_{t}=\frac{d\sigma }{d\varepsilon }=\;\alpha \cdot \varepsilon +{\;E}_{0}$$

where E_t_ (= dσ/dε) is the tangent stiffness at a given value of ε, E_0_ is the initial stiffness (at zero strain), and α is the stress–strain curvature coefficient, respectively.

The second-order polynomial relation between stress and strain is derived by integrating the above equation with respect to ε (for intercept at origin):

(5) $$\sigma \;=\frac{1}{2}\cdot \alpha \;\cdot \varepsilon^{2}+E_{0}\cdot \varepsilon$$

Between any two points on the second-order polynomial stress–strain curve, the relationship between relative increment in stress $\left(\sigma \;\%=\frac{\sigma_{2}-\sigma_{1}}{\sigma_{1}}\cdot 100\right)$ and relative increment in strain $\left(\varepsilon \;\%=\frac{\varepsilon_{2}-\varepsilon_{1}}{\varepsilon_{1}}\cdot 100\right)$ can be expressed as:

(6) $$\frac{\sigma \;\%}{\varepsilon \;\%}=\frac{\;\frac{1}{2}\cdot \alpha \;\cdot \left(\varepsilon_{1}+\varepsilon_{2}\right)+E_{0}}{\frac{1}{2}\cdot \alpha \;\cdot \varepsilon_{1}+E_{0}}$$

For a straight line (α = 0), Equation (6) reduces to$\;\frac{\sigma \;\%}{\varepsilon \;\%}=1$, i.e., the relative increment in stress and strain is the same. Equation (6) will be used in Section 3.2. to calculate relative increment in failure stress based on the determined relative increments in strain to failure.

In the present study, the experimental stress–strain curves were fitted with this second-order polynomial equation, and the fitted curves were used as practical operational stress–strain curves. Initial stiffness (E_0_) at zero strain was determined based on the fitted stress–strain curves using Equation (5). This method of stiffness determination takes into account the shape of the whole stress–strain curve. It overcomes the challenges of not well-defined stress–strain curves at the start of test, and the non-linear stress–strain behavior of carbon composites.

**Figure 5:**
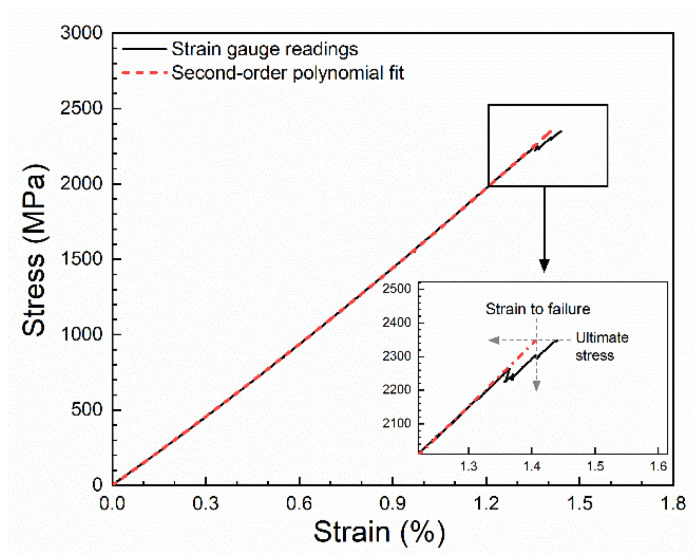
Example of experimental stress–strain curve and second-order polynomial fit showing the approach used to determine strain to failure.

As exemplified in Figure 5, some of the experimental stress–strain curves show serrations at the end of the curves, observed for both unprotected and protected test specimens. The reason of such small serrations could be due to various reasons like local failures in the specimen, slight slippage, gauge separation, or delamination. Therefore, due to these cases, a practical operational definition of failure stress and strain to failure is needed. As shown in Figure 5, failure stress was obtained as the measured ultimate stress (at maximum load). Then, strain to failure was obtained by extrapolating the fitted line to the ultimate stress. Based on the results of 36 test specimens (unprotected and protected carbon composites), the average corrected strain to failure ($\varepsilon_{\mathrm{corrected}}^{\mathrm{failure}}$) was lowered by 0.02% compared to measured strain to failure ($\varepsilon_{\mathrm{measured}}^{\mathrm{failure}}$); $\varepsilon_{\mathrm{measured}}^{\mathrm{failure}}-\varepsilon_{\mathrm{corrected}}^{\mathrm{failure}}\;\leq 0.02\;\%$. Therefore, this correction method had an extremely small influence on the determined strain to failure value.

#### 2.5.2. Back-Calculation of Stresses

During tensile testing of the protected carbon composite (G/C/G), the strain in each layer can be assumed equal to the measured strain for the whole composite due to the parallel arrangement of layers. Therefore, the corresponding stresses in each layer follow the rule of mixture relationships with the measured stress of the composite.

The stress in the carbon composite layer in the protected carbon composite (G/C/G), at a given value of strain, can be back-calculated as follows:

(7) $$\sigma_{C}=\left(\sigma_{G/C/G}-{\;\sigma }_{G}\cdot V_{G}-\sigma_{A}\cdot V_{A}\right)/V_{C}$$

where σ_C_, σ_G/C/G_, σ_G_, and σ_A_ are stresses in carbon composite layer, protected carbon composite, glass composite layer, and adhesive layer, respectively. Moreover, V_G_, V_A_, and V_C_ are volume fractions of glass composite, adhesive, and carbon composite in the protected carbon composite, respectively.

The stress values in the glass composite layers (σ_G_) were assumed similar to stress values in the tested unprotected glass composites (G). The experimental stress–strain curves of unprotected glass composites (G) were fitted with the second-order polynomial (Equation (5)).

**Figure 6:**
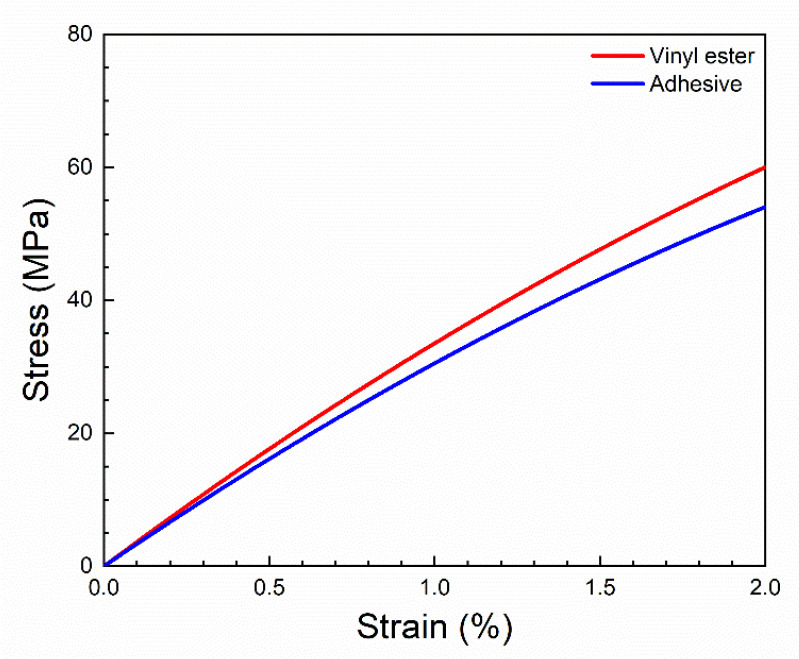
Established stress–strain curves of epoxy adhesive and vinyl ester matrix, shown in the relevant strain range. See approach in Appendix A.

The stress values in the adhesive layers (σ_A_) were found from an established stress–strain curve of epoxy adhesive, see Figure 6. The curve was established using the second-order polynomial (Equation (5)) based on values of failure stress, strain to failure, and stiffness found in the manufacturer’s datasheet. The approach is summarized in Appendix A.

For the carbon composite, using the assumption of equal strain, the stress of the carbon fibres, at a given value of strain, was back-calculated as follows:

(8) $$\sigma_{f}=\left(\sigma_{C}-{\;\sigma }_{m}\cdot V_{m}\right)/V_{f}$$

where σ_f_, σ_C_, and σ_m_ are stresses in carbon fibres, carbon composite and matrix, respectively. Moreover, V_m_ and V_f_ are volume fractions of matrix and fibres, respectively. The stress values in the matrix (σ_m_) were found with a similar approach as used above for the adhesive layer.

### 2.6. Single Fibre Tensile Testing

Single carbon fibres of the same type as used by Fiberline Composites A/S to manufacture the pultruded carbon composite were tensile tested. This was performed using a semi-automated single fibre testing machine (TexTechno, Favimat+, Moenchengladbach, Germany), with a built-in vibroscope system to determine the fibre cross-sectional area. For each tested fibre, based on the measured resonance frequency, the fibre linear density (T, g/1000 m) was determined. The fibre cross-sectional area (A = T/ρ) was then determined using the measured density of the carbon fibres (ρ, 1.77 g/cm^3^). The carbon fibres were tested with three different gauge lengths (30, 40, and 50 mm), and with a sample size of 150 for each gauge length.

## 3. Results and Discussion

### 3.1. Analysis of Stress–strain Curves

The measured volume fractions, V_C_, V_G_, and V_A_, in the protected carbon composites (G/C/G) were 30–31%, 67–68%, and 1–3%, respectively, as shown in Table 1. The measured values for each of the three specimen geometries are used in the further analysis to back-calculate the stress–strain curves for the carbon composite.

**Figure 7:**
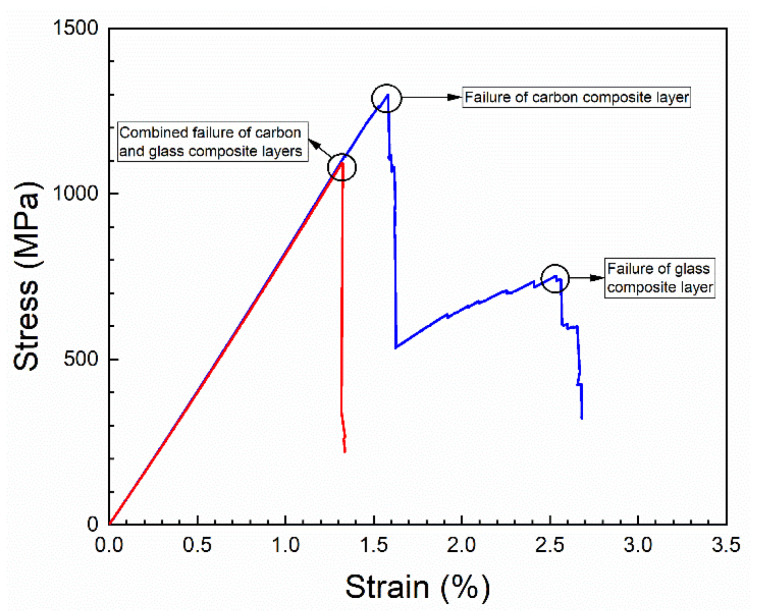
Representative stress–strain curves showing the two failure modes in the protected carbon composites: single failure mode (red) and double failure mode (blue).

Two different failure modes were observed for the protected composites, as shown by the stress–strain curves in Figure 7:

- Single failure mode, where failure of the central carbon composite layer is leading to immediate failure of the protective glass composite layers.
- Double failure mode, where failure of the central carbon composite layer is leading to a drop of load, and the load is then carried by the protective glass composite layers until their failure at a later stage of the tensile test.

The occurrence of two different failure modes indicates that the volume fraction of carbon composite in the protected carbon composites is in the vicinity of the so-called threshold volume fraction, which defines the transition between multiple fracture and single fracture in the simple model of strength of unidirectional composites [20]. In the present study, by using the measured values of stiffness and strain to failure of the unprotected carbon and glass composites, the threshold volume fraction is calculated to be 21%. This threshold value is lower than the measured volume fraction of carbon composites of about 30–31%. Thus, only single fracture should therefore be expected according to the simple strength model. However, the failure situation is not that simple, but has a more complex nature. Failure also depends on interface toughness and absolute thickness of the layers [21], not taken into consideration in the simple strength model. In the present study, for the observed two failure modes, the first drop of stress of the stress–strain curves is assumed to represent failure of the carbon composite layer. No difference in strain to failure was found between the two failure modes. In the further presentation of stress–strain curves, the curves are shown only until their first major drop of stress, representing failure of the carbon composite layer.

**Figure 8:**
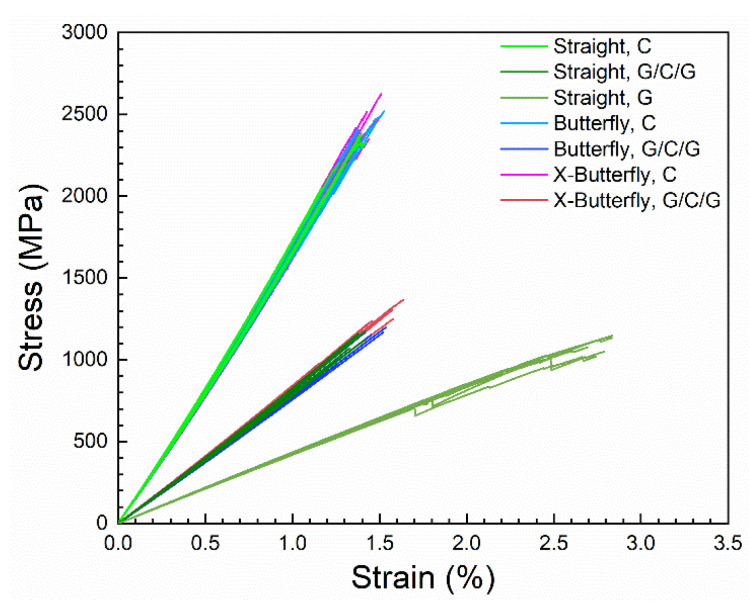
The seven groups of experimental stress–strain curves. The datasets is available in reference [22].

Figure 8 shows the experimental stress–strain curves of the present study. The figure shows the seven groups of stress–strain curves: unprotected and protected carbon composites (C and G/C/G) with three different specimen geometries (straight-sided, butterfly, and X-butterfly), and the unprotected glass composites (G, straight-sided). As expected, on a mere comparative basis, the carbon composites (C) are showing high stiffness, high failure stress, and low failure strain. The glass composites (G) are showing low stiffness, low failure stress, and high failure strain. The protected carbon composites (G/C/G) are showing intermediate stiffness, low failure stress similar to the glass composites, and low failure strain similar to the carbon composites. The determined mechanical properties will be thoroughly analyzed in Section 3.2 and Section 3.3 for the effect of specimen geometry and protection.

**Figure 9:**
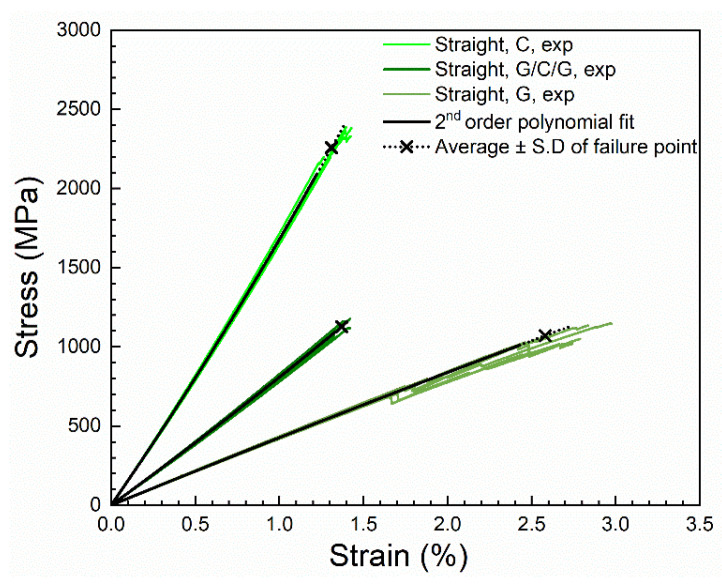
Experimental and second-order polynomial fitted stress–strain curves of straight-sided specimens of unprotected and protected carbon composites, and unprotected glass composites. The single fitted curve for each type of composite represents an average of the individually fitted curves.

Figure 9 shows the experimental stress–strain curves of straight-sided specimens, together with the average of the second-order polynomial fitted curves. On the fitted curves, a cross (×) represents the average failure point, i.e., the average of failure stress, and the average of strain to failure of the tested specimens. The dotted part at the end of the fitted curves represents the standard deviation of the failure stress and the strain to failure, i.e., there is 68% probability that the failure point will lie on the dotted line. It is found that this is a good way of representing the spread of data.

**Figure 10:**
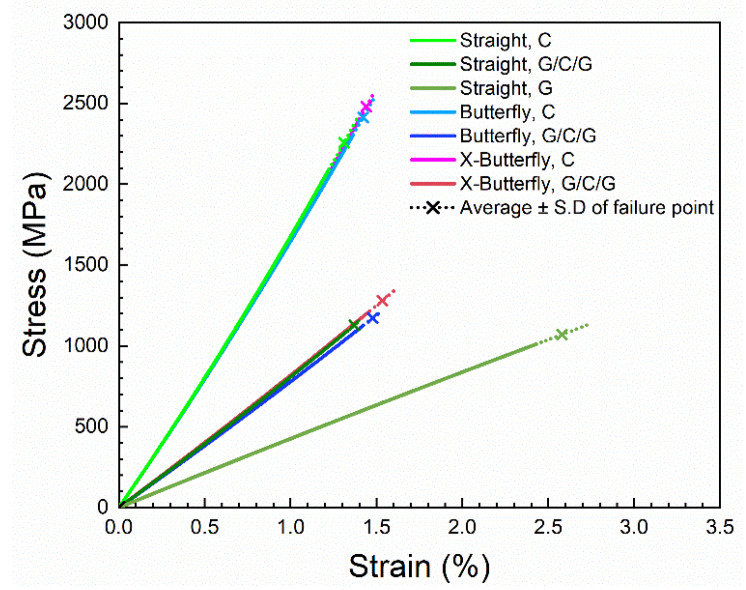
The seven groups of fitted stress–strain curves.

As exemplified in Figure 9 for the straight-sided specimens, an excellent agreement is found between the experimental stress–strain curves and the fitted curves. Accordingly, in the further analysis, the fitted stress–strain curves will be used. Figure 10 shows the fitted curves of the seven groups of stress–strain curves.

**Figure 11:**
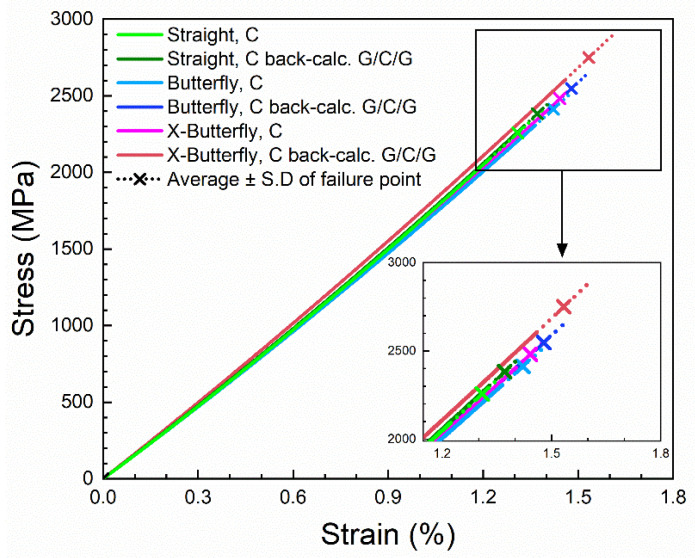
Back-calculated stress–strain curves of carbon composite layer from protected carbon composites, together with stress–strain curves of unprotected carbon composites.

The contribution of the carbon composite layer in the protected carbon composites was back-calculated by the use of Equation (7). The stress–strain curves of the unprotected carbon composites are shown together with the back-calculated stress–strain curves for the carbon composite layer in Figure 11. For all curves, the values of the second-order polynomial parameters of initial stiffness (E_0_) and curvature coefficient (α) are similar in the ranges 153–158 GPa and 2000–2800 GPa, respectively. Thus, a good agreement between the stress–strain curves is found (only the failure points differ between them as will be analyzed next), and this validates the applied analysis of stress–strain curves, as well as the method of back-calculation. The back-calculated stress–strain curves for the carbon composite layer lay the foundation of the forthcoming analysis on the effect of specimen geometry and protection.

### 3.2. Effect of Specimen Geometry

As shown in Table 2, the determined initial stiffness of unprotected carbon composites with different specimen geometries (straight-sided, butterfly, and X-butterfly) were found to be a coinciding value at 153 GPa. This validates the experimental methods since a change in specimen geometry should have no effect on the determined stiffness. For the protected carbon composites, a small difference in stiffness in the range 75–79 GPa was found between the specimen geometries, which, however, can be expected due to the small difference in their composition (see Table 1). This will be utilized in the later analysis of the contribution of the carbon composite layer.

**Figure 12:**
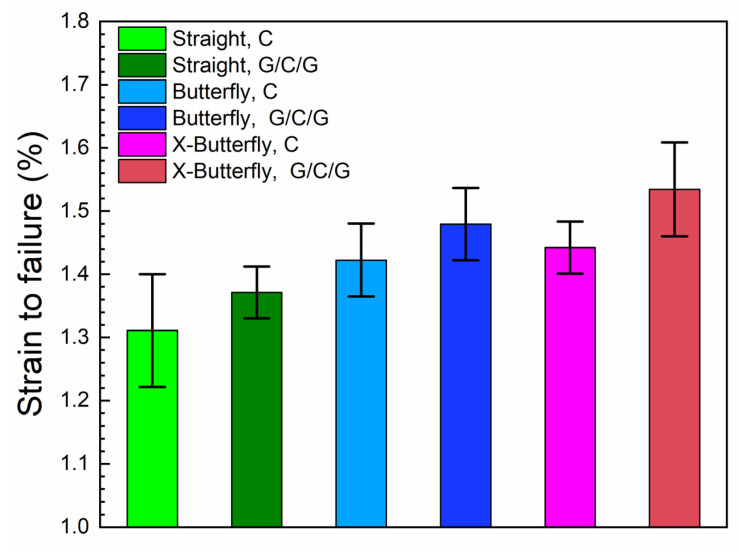
Bar graph representing strain to failure (%) for different specimen geometries of unprotected and protected carbon composites.

The effect of specimen geometry on the failure point is evaluated by comparing the strain to failure of the composites. As shown in Table 3, for unprotected carbon composites, the strain to failure for straight-sided, butterfly, and X-butterfly specimens are 1.31, 1.42, and 1.44%, respectively. Comparing butterfly specimens to straight-sided ones, the effect of changing specimen geometry leads to a relative increment in strain to failure of 8.4% (= (1.42 − 1.31)/1.31). For X-butterfly specimens, the strain to failure is furthermore increased with a relative increment of 9.9% (= (1.44 − 1.31)/1.31). Thus, the use of the butterfly specimens leads to a significant increase in strain to failure, compared to the straight-sided specimens. The use of elongated X-butterfly specimens, instead of standard butterfly specimens, leads to a slight increase in the average strain to failure, but with overlapping stdvs. A similar effect of specimen geometry can be seen for the protected carbon composites, as also shown in Table 3. The results of strain to failure for the different specimen geometries are also presented in a bar graph (see Figure 12) for better visualization of the differences.

As clearly shown by the results in Table 3, the *straight-sided* specimens are not able to fully realize the maximum strain to failure of unidirectional composites. It is well documented that a stress concentration region exists at the start of the end-tabs, which is increasing the susceptibility of the material to fail in that region. De Baere et al. [6] reported a stress concentration factor of 1.27 for straight-sided carbon/PPS composite specimens with straight-edged end-tabs of glass/epoxy composite. Korkiakoski et al. [13] performed a finite element analysis of straight-sided (ISO 527-5:2009) UD glass/epoxy specimens and found a stress concentration factor of 1.32 at the start of the end-tabs.

**Figure 13:**
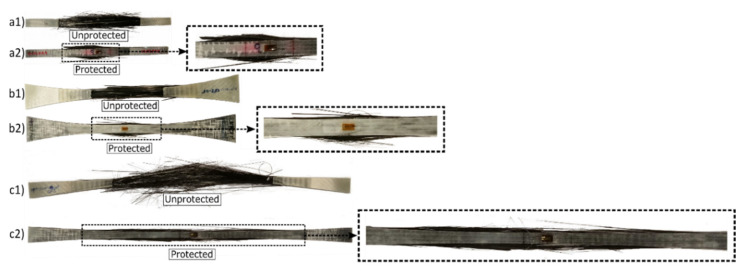
Examples of failed unprotected and protected carbon composite specimens: (**a1**,**a2**) straight-sided, (**b1**,**b2**) butterfly, and (**c1**,**c2**) X-butterfly.

The use of *butterfly-shaped* specimens with low curvature together with long tapered end-tabs is known to reduce the risk of failure at the end-tabs. Korkiakoski et al. [13] also performed finite element analysis of butterfly-shaped UD glass/epoxy specimens, and reported a stress concentration factor of only 1.02 at the start of the tapered end-tabs. Similarly, De Baere et al. [9] used dumbbell shaped carbon/PPS specimens, and reported significantly higher strength values than for straight-sided specimens. However, such non-straight-sided specimens are known to split in the longitudinal direction from the edges of the curved area [9]; this damage mode is known as longitudinal splitting. It occurs due to low shear resistance of the UD composites and can cause premature failure. In the present study, such longitudinal splitting cracks were indeed observed on the surface of the outer glass composite layer of protected butterfly and X-butterfly specimens. Likewise, such longitudinal splitting cracks are also expected to occur in the unprotected butterfly and X-butterfly specimens, although difficult to detect visually due to the extensive broom-like failure between the end-tabs (see Figure 13). Longitudinal splitting did not seem to influence the final failure, since failure always occurred in the region of the gauge section (see later discussion in Section 3.3). So, based on the observations in the present study, longitudinal splitting was not found to be the cause of final failure of the specimens.

The *X-butterfly* specimens are designed with a lower curvature, compared to the butterfly ones, to minimize shear stresses at the edges of the specimen, and to have a smoother stress field [13]. Thus, as expected, a slight improvement was observed in the strain to failure of the specimens with X-butterfly geometry.

It can be speculated that the non-identical tested materials volume of the three different specimen geometries in itself may result in variation of strain to failure due to a size effect; as the probability of finding a defect increases with materials volume as typically analyzed with the weakest link Weibull model [23]. Wisnom et al. [24] analyzed the size effect on strength of unidirectional carbon/epoxy composites, and a 14% reduction in failure stress was measured for an increase in materials volume by a factor of 500. In the present study, considering only the gauge section of the specimens, the materials volume of the straight-sided, butterfly, and X-butterfly specimens are 4580, 1720, and 920 mm^3^, respectively. Considering the whole section of the specimens between the grips, the tested materials volume of the straight-sided, butterfly and X-butterfly specimens are 4580, 9380, and 8080 mm^3^, respectively.

In straight-sided specimens, a constant stress state exists due to the constant material volume throughout the whole section between the grips. However, in butterfly and X-butterfly specimens, the stress state varies along the length, with highest stress state occurring in the central rectangular section. A size effect model was established based on Weibull’s [23] derivation of probability of failure for non-uniform stress state. The established model calculates the ratio of stresses in the gauge section of the two specimen geometries (butterfly and straight-sided) for the same probability of failure. For the butterfly specimens, dimensions and stresses are integrated over the tested material’s volume. Details of the model will be presented elsewhere [25]. For a Weibull modulus of 41, which previously has been found by Wisnom et al. for UD carbon composites [24], the established model predicts 1.3 and 2.5% relative increment in failure stress for the butterfly and X-butterfly specimens compared to the straight-sided ones. These values are relatively small compared to the determined relative increment in failure stress of 9.3 and 10.9% for unprotected butterfly and X-butterfly specimens, respectively. These values of relative increment in failure stress were calculated using Equation (6) based on the determined relative increments in strain to failure of 8.4 and 9.9%, as presented above. Altogether, it is found that the potential size effect phenomenon is relatively small compared to the effect of specimen geometry.

### 3.3. Effect of Specimen Protection

The strain to failure for unprotected and protected carbon composites for each of the three different specimen geometries are shown in Table 3, and in the bar graph in Figure 12 for better visualization of the differences. The results are consistently showing higher strain to failure in the protected carbon composites compared to the unprotected carbon composites. The relative increment of strain to failure due to specimen protection is similar in the range of 4–6% for the three specimen geometries. The largest difference is seen for the X-butterfly specimens where the strain to failure is increased from 1.44 to 1.53%, giving a relative increment of 6.2% (= (1.53 − 1.44)/1.44).

In the protected carbon composites, the outer glass composite layers protect the sandwiched carbon composite from damage of the grips, and thereby acting similar to the end-tabs of the unprotected specimens. In addition, the continuous outer layers ensures that there is no sudden (or gradient) change in the thickness direction, as it is the case for straight edged end-tabs (or tapered end-tabs). Avoiding such changes in the thickness direction ensures that there are no stress concentrations due to stress singularity, which previously have been reported for UD composite specimens at the start of the end-tabs [13]. For interlayer glass/carbon/glass composite specimens, Cźel et al. [14] performed linear elastic finite element modelling to analyze stress concentrations around straight edged end-tabs. So, those specimens had both continuous protective layers (glass composite) and end-tabs. It was demonstrated that the stress concentrations at the start of the end-tabs do not affect the strain in the central carbon layer. Regions of stress concentrations occurred only in the protective glass composite layers. In the protected carbon composite specimens of the present study, with no end-tabs, regions of stress concentrations will occur at the edges of the grips. Here, as it was demonstrated in the study by Cźel et al., the outer glass composite layers will serve to restrict stress concentrations through applying a sufficient thickness of the protective layers. In addition to restricting stress concentrations at the grips, the outer glass composite layers may also help restricting growth of longitudinal splitting cracks in the curved regions of the butterfly specimens, and thereby reducing the contribution of longitudinal splitting to the final failure of the specimens. This benefit of protective layers needs to be addressed in future studies designed specifically to accurately detect and analyze the initiation and growth of longitudinal splitting cracks.

Figure 13 shows representative images of failed unprotected and protected carbon composite specimens. For all specimens, a broom-like failure can be observed. Such failure mode is usually observed for UD composites due to the relative low fibre/matrix interface strength causing a high degree of fibre debonding before fibre failure [26]. As a result, extensive fibre splitting is observed upon final failure of the composite [26]. In the unprotected specimens, some broken end of fibres near the start of the end-tabs can be observed. This can be attributed to the stress concentration regions located at the start of end-tabs, as discussed above. For the unprotected specimens, the widespread failure region makes it difficult to know if the failure is valid, i.e., the specimens should fail centrally in the gauge section rather than near the end-tabs and the grips. In the protected specimens, however, for all three geometries, failure can be observed to be more restricted in the gauge section, as shown in the zoomed images in Figure 13. This observation indicates that the protective glass composite layers help promote failure of the carbon composite in the gauge section. The protective layer makes it easier to judge the validity of failure.

### 3.4. Combined Effect of Specimen Geometry and Protection

By using the unprotected straight-sided specimens as basis, the effect of changing to X-butterfly-shaped specimens is a relative increment in strain to failure of 9.9%, as already presented earlier. If protective layers are used, the strain to failure is furthermore increased with a relative increment of 6.9%, as also presented earlier. Thus, the combined effect of changing geometry and protecting the specimen is a relative increment of 16.8%. This represents a significant improvement in failure properties of the carbon composites, and will have direct implications for the design of structural applications, e.g., by reducing the amount of materials needed for certain load cases.

**Figure 14:**
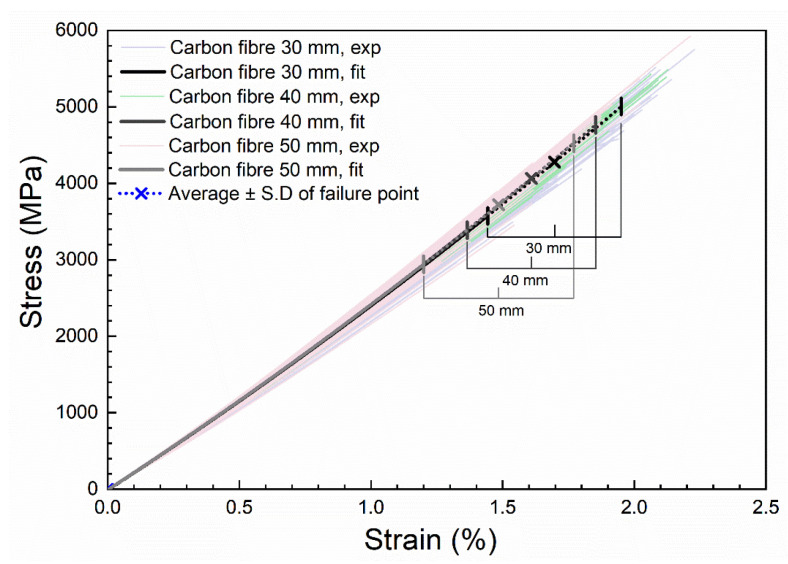
Experimental and fitted stress–strain curves of single carbon fibres tested with gauge lengths of 30, 40, and 50 mm. The datasets is available in reference [29].

In the present study, stress–strain curves of single carbon fibres were measured and compared to back-calculated stress–strain curves from the carbon composites. Figure 14 shows the experimental stress–strain curves of single carbon fibres for 30, 40, and 50 mm gauge lengths, together with the second-order polynomial fitted curves. The experimental stress–strain curves show the scattered trend typically observed for single fibres [27,28]. An excellent agreement, however, can be observed between the fitted curves for the three-gauge length; the curves are precisely falling on top of each other. The determined strain to failure of the carbon fibres for the three different gauge lengths are summarized in Table 4. It can be seen that the average strain to failure is highly dependent on the gauge length. At short gauge lengths, strain to failure values are higher, and they decrease with an increase of the gauge length.

**Figure 15:**
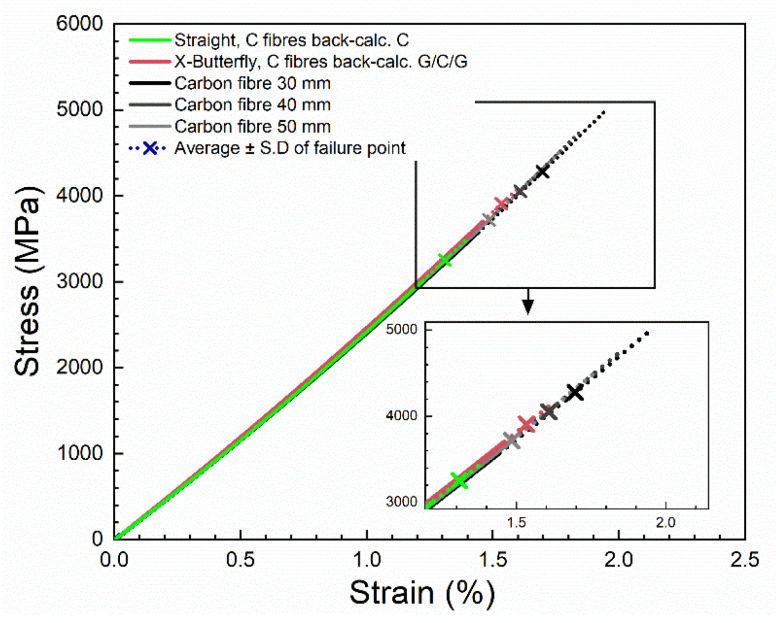
Fitted stress–strain curves of single carbon fibres and back-calculated stress–strain curves of carbon fibres from composites.

Figure 15 shows the fitted stress–strain curves of single carbon fibres, together with back-calculated stress–strain curves of carbon fibres (from fitted stress–strain curves of composites, using a measured fibre volume fraction of 67%). For all curves, the values of the second-order polynomial parameters of initial stiffness (E_0_) and curvature coefficient (α) are similar in the ranges 224–234 GPa and 3000–4300 GPa, respectively. Thus, a good agreement between the stress–strain curves are found. It demonstrates the efficacy of the second-order polynomial fitting method for back-calculation of fibre properties, for comparison in terms of stiffness and curvature coefficient values.

The observed increasing trend in the measured strain to failure of the carbon composites (by changing geometry and protection) suggests that the *actual strain to failure* of the carbon composites is getting closer to be realized.

## 4. Conclusions

This paper presents advances in tensile testing of unidirectional carbon fibre composites by using an improved specimen type by combining specimen geometry and specimen protection, together with a robust data analysis method of second-order polynomial fitting. Results of tensile testing of unprotected and protected carbon fibre composite specimens with three different specimen geometries (straight-sided, butterfly, and X-butterfly) have been presented and analyzed.

The central finding of the study is that the protected X-butterfly specimens led to an improvement of 17% on the measured strain to failure compared to rectangular-shaped specimens based on widely used standards like ISO 527-5:2009 and ASTM D3039. This represents a significant improvement in measured failure properties, and will have direct implications for the design of structural applications with carbon composites.

Additional findings of the study are hereby presented:

- An excellent agreement is found between the experimental stress–strain curves and the second-order polynomial fitted curves. Accordingly, it is demonstrated that the fitted stress–strain curves can be used as practical operational curves for further analysis.
- A good agreement between back-calculated stress–strain curves of protected carbon composites and measured stress–strain curves of unprotected carbon composites is found. Similarly, a good agreement is found between back-calculated stress–strain curves of carbon fibres and measured stress–strain curves of single carbon fibres. Only the failure points differ between the curves. Altogether, this validates the efficacy of the applied analysis of stress–strain curves and method of back-calculation.
- Initial stiffness (E_0_) that takes into account the shape of the whole stress–strain curve was determined based on the practical operational stress–strain curves. Initial Stiffness of unprotected carbon composites with the three different specimen geometries were found to be identical at 153 GPa. This is expected since a change in specimen geometry should have no effect on the determined stiffness.
- Comparing butterfly specimens to straight-sided ones, the effect of changing specimen geometry was an increase in strain to failure from 1.31 to 1.42%. For the elongated X-butterfly specimens, strain to failure was furthermore increased to 1.44%.
- It can be speculated that the non-identical tested materials volume of the three different specimen geometries in itself may result in variation of strain to failure due to a materials size effect. Based on Weibull model calculations, it is found that the potential size effect phenomenon is relatively small compared to the effect of specimen geometry.
- The protected carbon composites showed higher strain to failure compared to the unprotected carbon composites. The relative increment of strain to failure was in the range of 4–6% for the three specimen geometries. The largest difference was seen for the X-butterfly specimens where the strain to failure was increased from 1.44 to 1.53%.
- An extensive broom-like failure mode was observed for all specimens. In the protected specimens, however, failure was observed to be more restricted in the gauge section. This observation indicates that the protective layers help promote failure of the carbon composite in the gauge section.

The observed increasing trend in the measured strain to failure of the carbon composites (by changing geometry and protection) suggests that the actual strain to failure of the carbon composites is getting closer to be realized. The obtained largest value of 1.53% for the protected X-butterfly specimens is likely still to be an underestimation of the actual value. Thus, there is a scope of future research to evaluate the actual strain to failure of unidirectional composites.

## Figures and Tables

**Table 1:** Measured composite volume fractions (%) in the three specimen geometries of the protected carbon composites (G/C/G).

Specimen Geometries	Composite Volume Fractions		
	V_C_	V_G_	V_A_
Straight	31.0 ± 0.01	66.6 ± 0.2	2.4 ± 0.2
Butterfly	29.6 ± 0.01	67.4 ± 0.2	3.0 ± 0.3
X-Butterfly	31.1 ± 0.01	67.6 ± 0.2	1.3 ± 0.2

**Table 2:** Initial stiffness (GPa) of unprotected (C) and protected (G/C/G) carbon composites with three specimen geometries.

Carbon Composite	Specimen Geometry		
	Straight-Sided	Butterfly	X-Butterfly
Unprotected, C	153 ± 3	153 ± 3	153 ± 4
Protected, G/C/G	77 ± 2	75 ± 1	79 ± 1

**Table 3:** Strain to failure (%) of unprotected (C) and protected (G/C/G) carbon composites with three specimen geometries.

Carbon Composite	Specimen Geometry		
	Straight-Sided	Butterfly	X-Butterfly
Unprotected, C	1.31 ± 0.09	1.42 ± 0.06	1.44 ± 0.04
Protected, G/C/G	1.37 ± 0.04	1.48 ± 0.06	1.53 ± 0.07

**Table 4:** Strain to failure (%) of single carbon fibres tested with different gauge lengths.

Gauge length (mm)	30	40	50
Strain to failure (%)	1.69 ± 0.25	1.60 ± 0.24	1.48 ± 0.28

**Figure A1:**
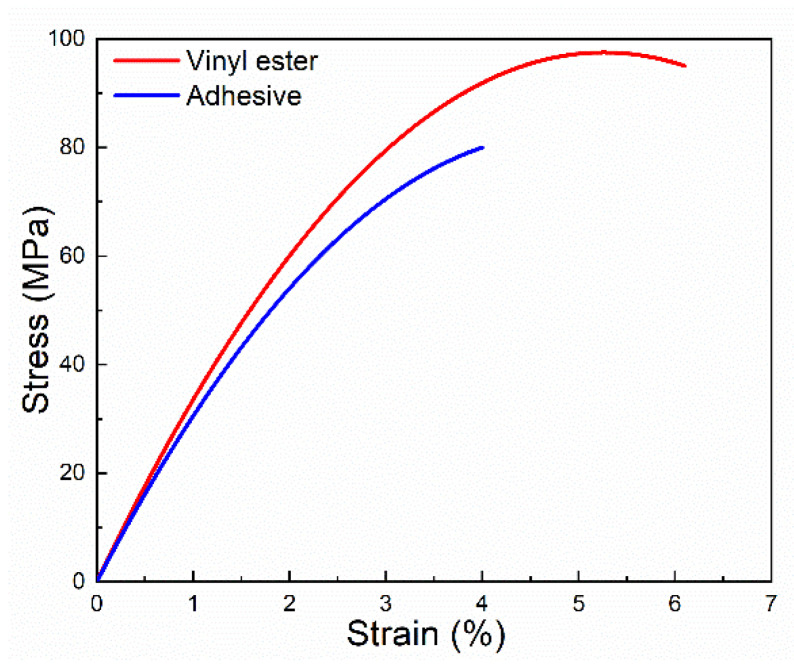
Estimated stress–strain curves of epoxy adhesive and vinyl ester matrix.

## Data Availability

The datasets are available in reference [22] and [29].

## Appendix A

Estimated stress–strain curves for adhesive and matrix.

If the stress–strain curve is approximated by a second order polynomial relation, the coefficients α (stress–strain curvature coefficient) and E_0_ (initial stiffness at zero strain) can be calculated based on knowledge of conventional stiffness (E_1_) and failure properties (σ_f_ and ε_f_).

A linear relation between E_1_ (conventional stiffness between strain 0.05–0.25%, with the mid value ε_1_ = 0.15%) and E_0_ is:

(A1) $$E_{1}=\alpha \;\cdot {\;\varepsilon }_{1}+{\;E}_{0}$$

The value of E_0_ can therefore be written as:

(A2) $$E_{0}={\;E}_{1}-\alpha \;\cdot \varepsilon_{1}$$

The second-order polynomial relation between stress and strain is:

(A3) $$\sigma \;=\frac{1}{2}\cdot \alpha \;\cdot \varepsilon^{2}+E_{0}\cdot \varepsilon$$

Using Equation (A2)

(A4) $$\sigma_{f}=\frac{1}{2}\cdot \alpha \;\cdot \varepsilon_{f}{}^{2}+(E_{1}-\alpha \;\cdot \varepsilon_{1})\cdot \varepsilon_{f}$$

where σ_u_ and ε_u_ are failure stress and strain to failure, respectively. The value of α can therefore be written as:

(A5) $$\alpha =\frac{\sigma_{f}-E_{1}\cdot \varepsilon_{f}}{\;\frac{1}{2}\cdot \varepsilon_{f}{}^{2}-\varepsilon_{1}\cdot \varepsilon_{f}}$$

Based on values of E_1_, σ_f_, and ε_f_ for the adhesive and the matrix from the manufacturer’s datasheet, values of α and E_0_ can be determined (using Equations (A5) and (A2)), see Table A1. Stress–strain curves can then be estimated (using Equation (A3)), see Figure A1.

**Table A1:** Values from the manufacturer’s datasheet for adhesive and matrix, and the determined values of E_0_ and α.

Material	$E_{1}\;(\mathrm{GPa})$	$\sigma_{f}\;(\mathrm{MPa})$	$\varepsilon_{f}\;(-)$	$\varepsilon_{1}\;(-)$	$E_{0}\;(\mathrm{GPa})$	α (GPa)
Epoxy adhesive	3.3	80	0.040	0.0015	3.4	−70.3
Vinyl ester matrix	3.6	95	0.061	0.0015	3.7	−70.4
